# Supplementary material for: The Evolution and Characterization of the RNA Interference Pathways in Lophotrochozoa
Source: Genome Biol Evol. 2024 May 7;16(5):evae098. doi: 10.1093/gbe/evae098 (PMC11114477; doi:10.1093/gbe/evae098)
Supplement: evae098_Supplementary_Data [file evae098_supplementary_data.zip › Supplementary_materials.zip!/selected_miRNAs/NoGe_selected_miRNAs/mirdeep_pdf_files/NMRB01000087.1_Notospermus_geniculatus_strain_Ushimado_scaffold87,_whole_genome_shotgun_sequence_3156.pdf]

Provisional ID	: NMRB01000087.1_Notospermus_geniculatus_strain_Ushimado_scaffold87,_whole_genome_shotgun_sequence_3156
Score total	: 26499.3
Score for star read(s)	: 3.9
Score for read counts	: 26490.6
Score for mfe	: 1.7
Score for randfold	: 0
Score for cons. seed	: 3
Total read count	: 51972
Mature read count	: 51722
Loop read count	: 0
Star read count	: 250

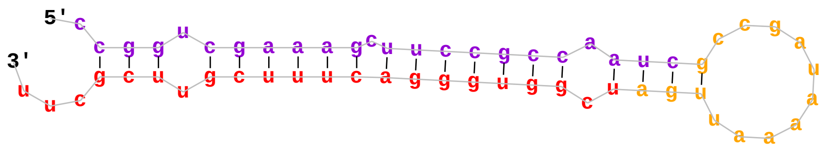

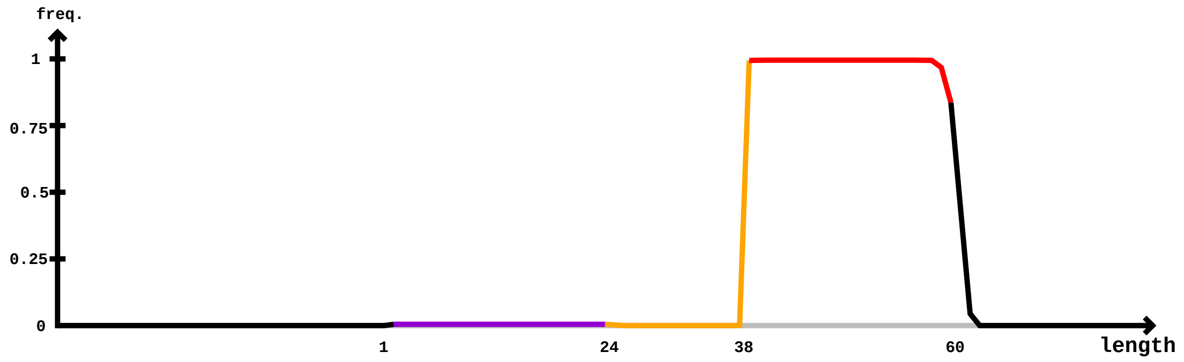

Star   Mature

```
5'- acgaagcgcaagagaacaaaccaccuguugcgcccggucgaaagcuuccgccaaucgccgauaaaaauugaucggugggacuuucguucgcuugugaugggugguagaucaac   -3'    obs
    acgaagcgcaagagaacaaaccaccuguugcgcccggucgaaagcuuccgccaaucgccgauaaaaauugaucggugggacuuucguucgcuugugaugggugguagaucaac          exp
    ...................(((((((((((((..(((.((((((.(((((((.((((...........)))).)))))))))))))).)))..)))))))))))))........   reads   mm   sample
    .................................ccggucgaaagcuuccgccaau..........................................................   3       0    Mm3
    .................................ccggucgaaagcuuccgccaauc.........................................................   18      0    Mm3
    .................................ccggucgaaagcuuccgccaaucA........................................................   1       1    Mm3
    .................................ccggucgaaagcuuccgccaaucU........................................................   2       1    Mm3
    ......................................................................Cucggugggacuuucguucgcu.....................   2       1    Mm3
    ......................................................................aucggugggacuuucguucgcuu....................   3       0    Mm3
    ......................................................................Cucggugggacuuucguucgcuu....................   2       1    Mm3
    .......................................................................ucggugggacuuucguuc........................   2       0    Mm3
    .......................................................................ucggugggacuuucguucg.......................   8       0    Mm3
    .......................................................................ucggugggacuuucguucU.......................   2       1    Mm3
    .......................................................................ucggugggacuuucguucA.......................   2       1    Mm3
    .......................................................................ucggugggacuuucguucgA......................   2       1    Mm3
    .......................................................................ucggugggacuuucguGcgc......................   1       1    Mm3
    .......................................................................ucggugggacuuucguucAc......................   1       1    Mm3
    .......................................................................ucggCgggacuuucguucgc......................   1       1    Mm3
    .......................................................................ucggugggacuuucguAcgc......................   1       1    Mm3
    .......................................................................ucggugggacuuucguuUgc......................   2       1    Mm3
    .......................................................................ucgguggUacuuucguucgc......................   1       1    Mm3
    .......................................................................ucggugggacuuucguuGgc......................   3       1    Mm3
    .......................................................................ucggugggacuuucguucgc......................   353     0    Mm3
    .......................................................................ucggugggacuuucguuAgc......................   2       1    Mm3
    .......................................................................ucggugggacuuucguucgU......................   3       1    Mm3
    .......................................................................ucggugggacCuucguucgc......................   2       1    Mm3
    .......................................................................ucggugggacAuucguucgc......................   1       1    Mm3
    .......................................................................ucggCgggacuuucguucgcu.....................   1       1    Mm3
    .......................................................................ucggugggCcuuucguucgcu.....................   1       1    Mm3
    .......................................................................ucggugggacuuucguuUgcu.....................   4       1    Mm3
    .......................................................................ucggugggacuuucguucgUu.....................   1       1    Mm3
    .......................................................................ucggugggacGuucguucgcu.....................   2       1    Mm3
    .......................................................................ucggugggacuuucguucgcC.....................   8       1    Mm3
    .......................................................................ucggugggacuuucguGcgcu.....................   4       1    Mm3
    .......................................................................ucggugggacuuucUuucgcu.....................   1       1    Mm3
    .......................................................................ucAgugggacuuucguucgcu.....................   2       1    Mm3
```

```
                                 Star                      Mature
acgaagcgcaagagaacaaaccaccuguugcgcccggucgaaagcuuccgccaaucgccgauaaaaauugaucggugggacuuucguucgcuugugaugggugguagaucaac
.......................................................................ucggugggacuuucgAucgcu.....................   1      1   Mm3
.......................................................................ucggugggacuuucguAcgcu.....................   4      1   Mm3
.......................................................................ucggugggacuuucguucgcA.....................   4      1   Mm3
.......................................................................ucggugggacuuucguuAgcu.....................   11     1   Mm3
.......................................................................ucggugggacuuucguCcgcu.....................   4      1   Mm3
.......................................................................ucggugggacuuucguucgcG.....................   29     1   Mm3
.......................................................................ucggugggacuuucguuGgcu.....................   4      1   Mm3
.......................................................................ucggugggacuuuUguucgcu.....................   1      1   Mm3
.......................................................................ucggugggGcuuucguucgcu.....................   1      1   Mm3
.......................................................................ucggugggacuuucguucgcu.....................   1401   0   Mm3
.......................................................................ucgguggUacuuucguucgcu.....................   1      1   Mm3
.......................................................................ucggugggUcuuucguucgcu.....................   1      1   Mm3
.......................................................................ucggugggacAuucguucgcu.....................   8      1   Mm3
.......................................................................ucggugNgacuuucguucgcuu....................   1      1   Mm3
.......................................................................uAggugggacuuucguucgcuu....................   3      1   Mm3
.......................................................................ucggugggacuuucguucgcuA....................   11     1   Mm3
.......................................................................ucggugggacuuuAguucgcuu....................   4      1   Mm3
.......................................................................ucggugggacGuucguucgcuu....................   3      1   Mm3
.......................................................................ucggugggacuuucguuUgcuu....................   16     1   Mm3
.......................................................................ucggugggCcuuucguucgcuu....................   1      1   Mm3
.......................................................................ucggugggacAuucguucgcuu....................   11     1   Mm3
.......................................................................ucUgugggacuuucguucgcuu....................   1      1   Mm3
.......................................................................ucggugggacuuucguAcgcuu....................   9      1   Mm3
.......................................................................ucggugggacuuucguuAgcuu....................   34     1   Mm3
.......................................................................ucgAugggacuuucguucgcuu....................   1      1   Mm3
.......................................................................ucgguggCacuuucguucgcuu....................   1      1   Mm3
.......................................................................ucggugggacuuucgCucgcuu....................   1      1   Mm3
.......................................................................ucggugggacuGucguucgcuu....................   1      1   Mm3
.......................................................................ucgguggUacuuucguucgcuu....................   1      1   Mm3
.......................................................................ucggugggUcuuucguucgcuu....................   1      1   Mm3
.......................................................................ucggugggacuuucguGcgcuu....................   11     1   Mm3
.......................................................................ucggugggacCuucguucgcuu....................   1      1   Mm3
.......................................................................ucggugggacuuucAuucgcuu....................   2      1   Mm3
.......................................................................ucggugggacuuucguucgUuu....................   1      1   Mm3
.......................................................................ucggugggacuuucguucgcuG....................   25     1   Mm3
.......................................................................ucggugggacuuucguuGgcuu....................   12     1   Mm3
.......................................................................ucgguNggacuuucguucgcuu....................   2      1   Mm3
.......................................................................ucggugggacuuucUuucgcuu....................   1      1   Mm3
.......................................................................Ncggugggacuuucguucgcuu....................   2      1   Mm3
.......................................................................ucgguUggacuuucguucgcuu....................   2      1   Mm3
.......................................................................ucggugggaGuuucguucgcuu....................   1      1   Mm3
.......................................................................ucggugggacuuucguucgcuC....................   1      1   Mm3
.......................................................................ucggugggacuuucguucgcGu....................   1      1   Mm3
.......................................................................ucggugggacuuucguucgcuu....................   4142   0   Mm3
.......................................................................ucggugggacuuucguCcgcuu....................   11     1   Mm3
.......................................................................ucggugAgacuuucguucgcuu....................   1      1   Mm3
.......................................................................ucAgugggacuuucguucgcuu....................   2      1   Mm3
.......................................................................ucgguAggacuuucguucgcuu....................   1      1   Mm3
.......................................................................ucggugggacuuuGguucgcuu....................   1      1   Mm3
.......................................................................ucggAgggacuuucguucgcuu....................   1      1   Mm3
.......................................................................ucggugggacuuCcguucgcuu....................   1      1   Mm3
.......................................................................ucggugggGcuuucguucgcuu....................   1      1   Mm3
.......................................................................ucggugggacuuucguucUcuu....................   1      1   Mm3
.......................................................................ucggugggacuuucguucgcCu....................   7      1   Mm3
.......................................................................uGggugggacuuucguucgcuu....................   1      1   Mm3
.......................................................................ucggugggacuuucguucgcuuC...................   3      1   Mm3
.......................................................................ucggugggacuuucguucgcuuU...................   1341   1   Mm3
.......................................................................ucggugggacuuucguucgcuuA...................   48     1   Mm3
.......................................................................ucggugggacuuucguucgcuug...................   4      0   Mm3
.......................................................................ucggugggacuuucguucgcuuCu..................   1      1   Mm3
.......................................................................ucggugggacuuucguucgcuuUu..................   362    1   Mm3
.......................................................................ucggugggacuuucguucgcuuAu..................   19     1   Mm3
........................................................................cggugggacuuucguucgcu.....................   3      0   Mm3
........................................................................cggugggacuuucguucgcuu....................   5      0   Mm3
........................................................................cggugggacuuucguucgcuuU...................   1      1   Mm3
........................................................................cggugggacuuucguucgcuuUu..................   2      1   Mm3
.........................................................................ggugggacuuucguucgcu.....................   1      0   Mm3
..........................................................................gugggacuuucguucgcuu....................   1      0   Mm3
...........................................................................ugggacuuucguucgcuu....................   1      0   Mm3

.................................ccggucgaaagcuuccgccaauc.........................................................   1      0   Fm3
```

```
                                Star                                Mature
acgaagcgcaagagaacaaaccaccuguugcgcccggucgaaagcuuccgccaaucgccgauaaaaauugaucggugggacuuucguucgcuugugaugggugguagaucaac
......................................................................aucggugggacuuucguucgcu.....................    1     0    Fm3
......................................................................aucggugggacuuucguucgcuu....................    4     0    Fm3
.......................................................................ucggugggacuuucguuc........................    2     0    Fm3
.......................................................................ucggugggacuuucguucg.......................    2     0    Fm3
.......................................................................ucggugggacuuucguucgU......................    3     1    Fm3
.......................................................................ucggugggacuuucguuAgc......................    6     1    Fm3
.......................................................................ucggugUgacuuucguucgc......................    1     1    Fm3
.......................................................................ucggugggacuuucguuGgc......................    1     1    Fm3
.......................................................................ucggugggacGuucguucgc......................    1     1    Fm3
.......................................................................ucggugggacuuucguucgc......................  564     0    Fm3
.......................................................................ucggugggacuuucguuUgc......................    2     1    Fm3
.......................................................................ucggugggacuuucguucgA......................    1     1    Fm3
.......................................................................ucggugggacuuucguGcgc......................    7     1    Fm3
.......................................................................ucggugggacCuucguucgc......................    1     1    Fm3
.......................................................................ucggugggUcuuucguucgc......................    1     1    Fm3
.......................................................................ucggugggacuuucguCcgc......................    2     1    Fm3
.......................................................................uUggugggacuuucguucgc......................    1     1    Fm3
.......................................................................ucggugggacGuucguucgcu.....................    1     1    Fm3
.......................................................................ucggugggacuuucguuAgcu.....................   23     1    Fm3
.......................................................................ucggugggacAuucguucgcu.....................    6     1    Fm3
.......................................................................ucggugggacuuucguAcgcu.....................   10     1    Fm3
.......................................................................ucgguggAacuuucguucgcu.....................    1     1    Fm3
.......................................................................ucggugggUcuuucguucgcu.....................    4     1    Fm3
.......................................................................ucggugggacuuucguGcgcu.....................    6     1    Fm3
.......................................................................ucggugggacuuucguucgcG.....................    2     1    Fm3
.......................................................................ucggugggacuuucUuucgcu.....................    1     1    Fm3
.......................................................................ucggugggacCuucguucgcu.....................    3     1    Fm3
.......................................................................ucggugggacuuucguucgcA.....................    5     1    Fm3
.......................................................................ucggugggacuuucguuUgcu.....................    5     1    Fm3
.......................................................................ucggugggacuCucguucgcu.....................    1     1    Fm3
.......................................................................ucggugggacuuuGguucgcu.....................    1     1    Fm3
.......................................................................ucgguggUacuuucguucgcu.....................    1     1    Fm3
.......................................................................uUggugggacuuucguucgcu.....................    1     1    Fm3
.......................................................................ucggugggacuuuUguucgcu.....................    1     1    Fm3
.......................................................................ucggugUgacuuucguucgcu.....................    1     1    Fm3
.......................................................................ucggugAgacuuucguucgcu.....................    1     1    Fm3
.......................................................................Ncggugggacuuucguucgcu.....................    3     1    Fm3
.......................................................................ucggugggacuuucguCcgcu.....................   10     1    Fm3
.......................................................................uAggugggacuuucguucgcu.....................    2     1    Fm3
.......................................................................ucggugggacuuucguucgcu..................... 2248     0    Fm3
.......................................................................ucggugggacuuucguuGgcu.....................    5     1    Fm3
.......................................................................ucggCgggacuuucguucgcu.....................    1     1    Fm3
.......................................................................ucggugggGcuuucguucgcu.....................    1     1    Fm3
.......................................................................ucggugggacuuucguGcgcuu....................    8     1    Fm3
.......................................................................ucggugggacAuucguucgcuu....................    8     1    Fm3
.......................................................................ucggugggGcuuucguucgcuu....................    2     1    Fm3
.......................................................................ucggugggacuuucguuGgcuu....................    7     1    Fm3
.......................................................................ucggugggacuuuAguucgcuu....................    1     1    Fm3
.......................................................................Ccggugggacuuucguucgcuu....................    1     1    Fm3
.......................................................................ucggugggacuuucUuucgcuu....................    1     1    Fm3
.......................................................................ucggugggacCuucguucgcuu....................    5     1    Fm3
.......................................................................ucggAgggacuuucguucgcuu....................    1     1    Fm3
.......................................................................Ncggugggacuuucguucgcuu....................    2     1    Fm3
.......................................................................ucCgugggacuuucguucgcuu....................    1     1    Fm3
.......................................................................ucggugUgacuuucguucgcuu....................    2     1    Fm3
.......................................................................ucggugggacuuucguucUcuu....................    3     1    Fm3
.......................................................................ucggugggacuuCcguucgcuu....................    1     1    Fm3
.......................................................................ucggugggacuuucguucgcuA....................   11     1    Fm3
.......................................................................ucggGgggacuuucguucgcuu....................    1     1    Fm3
.......................................................................ucggugggacuuuUguucgcuu....................    3     1    Fm3
.......................................................................ucUgugggacuuucguucgcuu....................    1     1    Fm3
.......................................................................ucggugggacuAucguucgcuu....................    1     1    Fm3
.......................................................................ucggugggacuuucguucgAuu....................    1     1    Fm3
.......................................................................ucggugggacuuucguucgUuu....................    2     1    Fm3
.......................................................................ucggugggacuuucguAcgcuu....................    6     1    Fm3
.......................................................................ucggugggacGuucguucgcuu....................    3     1    Fm3
.......................................................................ucggugggacuuucguCcgcuu....................   19     1    Fm3
.......................................................................uAggugggacuuucguucgcuu....................    3     1    Fm3
.......................................................................ucggugggacuuucguucgcuu.................... 3200     0    Fm3
.......................................................................ucgguggUacuuucguucgcuu....................    1     1    Fm3
```

Star **Mature**

acgaagcgcaagagaacaaaccaccuguugcgcccggucgaaagcuuccgccaaucgccgauaaaaauugaucggugggacuuucguucgcuugugaugggugguagaucaac			
....ucAgugggacuuucguucgcuu....................	4	1	Fm3
....ucggugggacuuucguuUgcuu....................	20	1	Fm3
....ucgUugggacuuucguucgcuu....................	1	1	Fm3
....ucgguggCacuuucguucgcuu....................	2	1	Fm3
....ucggugggacuGucguucgcuu....................	1	1	Fm3
....ucggugggacuuucAuucgcuu....................	1	1	Fm3
....ucggugggCcuuucguucgcuu....................	1	1	Fm3
....ucggugggacuCucguucgcuu....................	2	1	Fm3
....ucgguUggacuuucguucgcuu....................	2	1	Fm3
....ucggugggacuuucguuAgcuu....................	34	1	Fm3
....ucggugggacuuucguucgcuuA...................	9	1	Fm3
....ucggugggacuuucguucgcuuU...................	100	1	Fm3
....ucggugggacuuucguucgcuuUu..................	3	1	Fm3
....ucggugggacuuucguucgcuuAu..................	2	1	Fm3
....Uggugggacuuucguucgc......................	1	1	Fm3
....Uggugggacuuucguucgcu.....................	1	1	Fm3
....cggugggacuuucguucgcu.....................	5	0	Fm3
....cggugggacuuucguucgcuu....................	12	0	Fm3
....ggugggacuuucguucgc......................	2	0	Fm3
....ggugggacuuucguucgcu.....................	2	0	Fm3
....ggugggacuuucguucgcuu....................	1	0	Fm3
.................................ccggucgaaagcuuccgccaau..	2	0	Mm2
.................................ccggucgaaagcuuccgAcaauc...	1	1	Mm2
.................................ccggucgaaagcuuccgccaauc...	29	0	Mm2
.................................ccggucgaaaAcuuccgccaauc...	1	1	Mm2
.................................ccggucgaaagcuuccgUcaauc...	1	1	Mm2
.................................ccggucgaaagcuuccgccaaucgcc..	1	0	Mm2
....aucggugggacuuucguucgcuu...................	2	0	Mm2
....ucggugggacuuucguuU........................	1	1	Mm2
....ucggugggacuuucguuc........................	2	0	Mm2
....ucggugggacuuucguuAg.......................	1	1	Mm2
....ucggugggacGuucguucgc......................	1	1	Mm2
....ucggugggacuuucguuAgc......................	2	1	Mm2
....ucggugggacuuuUguucgc......................	1	1	Mm2
....ucggugggacuuucguucgc......................	102	0	Mm2
....ucggugggacuuucguuAgcu.....................	6	1	Mm2
....ucggugggacuuucguAcgcu.....................	1	1	Mm2
....ucggugggacCuucguucgcu.....................	1	1	Mm2
....ucggugggacuuucguuGgcu.....................	1	1	Mm2
....ucggugggGcuuucguucgcu.....................	4	1	Mm2
....ucggugggacuuucguGcgcu.....................	4	1	Mm2
....Ncggugggacuuucguucgcu.....................	2	1	Mm2
....ucggugggacuuucguucgcG.....................	24	1	Mm2
....ucggugggacuuucguuUgcu.....................	3	1	Mm2
....ucggugggUcuuucguucgcu.....................	2	1	Mm2
....ucggugggacuuucAuucgcu.....................	1	1	Mm2
....ucggugggacuuucguucgcu.....................	717	0	Mm2
....ucggugggacAuucguucgcu.....................	1	1	Mm2
....ucggugggacuuuUguucgcu.....................	1	1	Mm2
....ucggugggacuuucguucgAuu....................	1	1	Mm2
....ucggugggacuuucgCucgcuu....................	1	1	Mm2
....ucgguUggacuuucguucgcuu....................	2	1	Mm2
....ucggugggacuuuGguucgcuu....................	1	1	Mm2
....ucggugggacuuucguCcgcuu....................	15	1	Mm2
....ucggugggacuuucguuUgcuu....................	28	1	Mm2
....ucggugggacuuAcguucgcuu....................	1	1	Mm2
....ucggugggacAuucguucgcuu....................	8	1	Mm2
....ucggugAgacuuucguucgcuu....................	3	1	Mm2
....ucggugggacuuucguucgcuG....................	43	1	Mm2
....ucggugggacuuucguucgcuu....................	5291	0	Mm2
....ucggugggacuuucguAcgcuu....................	11	1	Mm2
....ucggugggacuuucguucgcCu....................	2	1	Mm2
....ucgguggUacuuucguucgcuu....................	3	1	Mm2
....ucggugggacuuucguucgcuC....................	4	1	Mm2
....Ncggugggacuuucguucgcuu....................	5	1	Mm2
....ucggugggacuuucguGcgcuu....................	13	1	Mm2
....ucggugggCcuuucguucgcuu....................	1	1	Mm2
....ucggCgggacuuucguucgcuu....................	2	1	Mm2
....ucggugggGcuuucguucgcuu....................	2	1	Mm2

```
                                Star                               Mature
acgaagcgcaagagaacaaaccaccuguugcgcccggucgaaagcuuccgccaaucgccgauaaaaauugaucggugggacuuucguucgcuugugaugggugguagaucaac
.......................................................................ucggugggaAuuucguucgcuu....................   1     1   Mm2
.......................................................................ucggugggUcuuucguucgcuu....................   7     1   Mm2
.......................................................................ucggugggacCuucguucgcuu....................   3     1   Mm2
.......................................................................ucggugggacuuuUguucgcuu....................   2     1   Mm2
.......................................................................ucggugggacuuucguuAgcuu....................   52    1   Mm2
.......................................................................ucggugCgacuuucguucgcuu....................   1     1   Mm2
.......................................................................ucggugggacuuucguucgcuA....................   17    1   Mm2
.......................................................................ucgguAggacuuucguucgcuu....................   2     1   Mm2
.......................................................................ucggugggacGuucguucgcuu....................   9     1   Mm2
.......................................................................ucggugUgacuuucguucgcuu....................   1     1   Mm2
.......................................................................uAggugggacuuucguucgcuu....................   1     1   Mm2
.......................................................................ucggugggacuuucguucgUuu....................   6     1   Mm2
.......................................................................ucAgugggacuuucguucgcuu....................   3     1   Mm2
.......................................................................ucggugggacuuucguuGgcuu....................   29    1   Mm2
.......................................................................ucggugggacuuuAguucgcuu....................   3     1   Mm2
.......................................................................ucggugggacuuucgAucgcuu....................   2     1   Mm2
.......................................................................ucggugggacuuucguucgcuuA...................   51    1   Mm2
.......................................................................ucggugggacuuucguucgcuuC...................   6     1   Mm2
.......................................................................ucggugggacuuucguucgcuuU...................   1794  1   Mm2
.......................................................................ucggugggacuuucguucgcuug...................   2     0   Mm2
.......................................................................ucggugggacuuucguucgcuuUu..................   399   1   Mm2
.......................................................................ucggugggacuuucguucgcuuCu..................   4     1   Mm2
.......................................................................ucggugggacuuucguucgcuuAu..................   8     1   Mm2
........................................................................cggugggacuuucguucgcuu....................   5     0   Mm2
........................................................................Uggugggacuuucguucgcuu....................   1     1   Mm2
.........................................................................ggugggacuuucguucgcuu....................   4     0   Mm2
..........................................................................gugggacuuucguucgcuu....................   1     0   Mm2

.................................ccggucgaaagcuuccgccaauc.........................................................   6     0   Mm1
......................................................................Cucggugggacuuucguucgcu.....................   3     1   Mm1
.......................................................................ucggugggacuuucguuUgc......................   2     1   Mm1
.......................................................................ucggugggacuuucguucgc......................   86    0   Mm1
.......................................................................ucggugggacuuucguGcgc......................   1     1   Mm1
.......................................................................ucggugggacuuucguAcgcu.....................   1     1   Mm1
.......................................................................ucggugggacuuucguuUgcu.....................   3     1   Mm1
.......................................................................ucggugggacuuucguuGgcu.....................   1     1   Mm1
.......................................................................ucggugggacuuucguGcgcu.....................   1     1   Mm1
.......................................................................ucggugggacuuucguucgcA.....................   6     1   Mm1
.......................................................................ucggugggacAuucguucgcu.....................   1     1   Mm1
.......................................................................ucggugggacuuucguucgcu.....................   293   0   Mm1
.......................................................................ucggugggacuuucguuAgcu.....................   1     1   Mm1
.......................................................................ucggugggacuuucguucgcuu....................   1068  0   Mm1
.......................................................................ucggugggacAuucguucgcuu....................   1     1   Mm1
.......................................................................ucggugggacuuucguuUgcuu....................   4     1   Mm1
.......................................................................Acggugggacuuucguucgcuu....................   1     1   Mm1
.......................................................................ucAgugggacuuucguucgcuu....................   1     1   Mm1
.......................................................................ucggugggacuuucguuGgcuu....................   1     1   Mm1
.......................................................................ucggugggacuuucguAcgcuu....................   6     1   Mm1
.......................................................................ucgguUggacuuucguucgcuu....................   2     1   Mm1
.......................................................................ucggugggacuuucguCcgcuu....................   6     1   Mm1
.......................................................................ucggugggacuuucguGcgcuu....................   2     1   Mm1
.......................................................................ucggugggacuuucguuAgcuu....................   8     1   Mm1
.......................................................................ucggugggacGuucguucgcuu....................   1     1   Mm1
.......................................................................ucggugggacuuucguucgcuuU...................   308   1   Mm1
.......................................................................ucggugggacuuucguucgcuuA...................   22    1   Mm1
.......................................................................ucggugggacuuucguucgcuuUu..................   20    1   Mm1
........................................................................cggugggacuuucguucgcuu....................   1     0   Mm1

.................................ccggucgaaagcuuccgcca............................................................   3     0   Fm1
.................................ccggucgaaagcuuccgccaau..........................................................   5     0   Fm1
.................................ccggucgaaagcuuccgccaauc.........................................................   140   0   Fm1
.................................Ucggucgaaagcuuccgccaauc.........................................................   1     1   Fm1
.................................ccggucgaaagcuuccgAcaauc.........................................................   2     1   Fm1
.................................ccggucgaaagcuuccgccaaucgccg.....................................................   1     0   Fm1
..................................cggucgaaagcuuccgccaaucgcc......................................................   2     0   Fm1
......................................................................aucggugggacuuucguucgcu.....................   3     0   Fm1
.......................................................................ucggugggacuuucguAc........................   1     1   Fm1
.......................................................................ucggugggacuuucguuc........................   9     0   Fm1
.......................................................................ucggugggacuuucguucU.......................   1     1   Fm1
.......................................................................ucggugggacuuucguucg.......................   7     0   Fm1
```

Star Mature

```
acgaagcgcaagagaacaaaccaccuguugcgcccggucgaaagcuuccgccaaucgccgauaaaaauugaucggugggacuuucguucgcuugugaugggugguagaucaac
.......................................................................ucggugggacuuucguucgc......................      37     0     Fm1
.......................................................................ucggugggacuuucguucgU......................      1      1     Fm1
.......................................................................uGggugggacuuucguucgcu.....................      1      1     Fm1
.......................................................................ucggugggacAuucguucgcu.....................      2      1     Fm1
.......................................................................ucUgugggacuuucguucgcu.....................      1      1     Fm1
.......................................................................ucggugggUcuuucguucgcu.....................      1      1     Fm1
.......................................................................ucggugggacuuucguucgcu.....................      1346   0     Fm1
.......................................................................ucggugggacGuucguucgcu.....................      1      1     Fm1
.......................................................................uAggugggacuuucguucgcu.....................      1      1     Fm1
.......................................................................ucgguAggacuuucguucgcu.....................      1      1     Fm1
.......................................................................ucggugAgacuuucguucgcu.....................      1      1     Fm1
.......................................................................ucggugggacuuucguucgcG.....................      58     1     Fm1
.......................................................................ucggugUgacuuucguucgcu.....................      1      1     Fm1
.......................................................................ucggugggacuuucguucgcC.....................      1      1     Fm1
.......................................................................ucggugggacuuucgAucgcu.....................      1      1     Fm1
.......................................................................ucggugggacuuuUguucgcu.....................      1      1     Fm1
.......................................................................ucggugggacuuucguuGgcu.....................      4      1     Fm1
.......................................................................ucggugggacuuucguuUgcu.....................      4      1     Fm1
.......................................................................ucggugggacuuucguCcgcu.....................      2      1     Fm1
.......................................................................ucgguggCacuuucguucgcu.....................      1      1     Fm1
.......................................................................ucggugggacuuucguuAgcu.....................      10     1     Fm1
.......................................................................ucggugggacCuucguucgcu.....................      2      1     Fm1
.......................................................................ucggugggacuuucguAcgcu.....................      2      1     Fm1
.......................................................................ucggugggacuuucguGcgcu.....................      2      1     Fm1
.......................................................................ucggugggacuuucguGcgcuu....................      38     1     Fm1
.......................................................................uGggugggacuuucguucgcuu....................      2      1     Fm1
.......................................................................ucggugggacuuucguucgcCu....................      3      1     Fm1
.......................................................................ucCgugggacuuucguucgcuu....................      3      1     Fm1
.......................................................................ucggugggacuuAcguucgcuu....................      2      1     Fm1
.......................................................................ucggugggacuuucguucgUuu....................      19     1     Fm1
.......................................................................uNggugggacuuucguucgcuu....................      2      1     Fm1
.......................................................................ucggugggacuuucguucgGuu....................      1      1     Fm1
.......................................................................ucggugggacuuuUguucgcuu....................      4      1     Fm1
.......................................................................uAggugggacuuucguucgcuu....................      8      1     Fm1
.......................................................................ucggugggacuuucguucgcuC....................      5      1     Fm1
.......................................................................ucggugggacuuucguucUcuu....................      4      1     Fm1
.......................................................................ucggugCgacuuucguucgcuu....................      4      1     Fm1
.......................................................................Acggugggacuuucguucgcuu....................      1      1     Fm1
.......................................................................ucggugggacuuucguucgcuG....................      133    1     Fm1
.......................................................................ucggCgggacuuucguucgcuu....................      1      1     Fm1
.......................................................................ucgguggGcuuucguucgcuu....................      9      1     Fm1
.......................................................................ucggugggacuuuAguucgcuu....................      1      1     Fm1
.......................................................................ucgAugggacuuucguucgcuu....................      6      1     Fm1
.......................................................................ucggugggaGuuucguucgcuu....................      1      1     Fm1
.......................................................................ucggugggacuuucguucgAuu....................      1      1     Fm1
.......................................................................ucgguCggacuuucguucgcuu....................      1      1     Fm1
.......................................................................ucgguUggacuuucguucgcuu....................      9      1     Fm1
.......................................................................ucgguAggacuuucguucgcuu....................      5      1     Fm1
.......................................................................ucgguggUacuuucguucgcuu....................      3      1     Fm1
.......................................................................Ccggugggacuuucguucgcuu....................      4      1     Fm1
.......................................................................ucggugggacuuuGguucgcuu....................      1      1     Fm1
.......................................................................ucggugggacuuucguCcgcuu....................      45     1     Fm1
.......................................................................ucggugggacAuucguucgcuu....................      16     1     Fm1
.......................................................................ucggugUgacuuucguucgcuu....................      3      1     Fm1
.......................................................................ucAgugggacuuucguucgcuu....................      4      1     Fm1
.......................................................................ucggugggacuuucguuAgcuu....................      113    1     Fm1
.......................................................................ucggugggacuuCcguucgcuu....................      1      1     Fm1
.......................................................................ucgguggCcuuucguucgcuu....................      5      1     Fm1
.......................................................................ucggugggacuuucguuUgcuu....................      60     1     Fm1
.......................................................................ucgCugggacuuucguucgcuu....................      2      1     Fm1
.......................................................................ucggugggacuuucguuGgcuu....................      55     1     Fm1
.......................................................................ucggugggacuuucguucgcuA....................      35     1     Fm1
.......................................................................ucggugggacCuucguucgcuu....................      15     1     Fm1
.......................................................................ucgguNggacuuucguucgcuu....................      1      1     Fm1
.......................................................................ucggGgggacuuucguucgcuu....................      1      1     Fm1
.......................................................................ucUgugggacuuucguucgcuu....................      6      1     Fm1
.......................................................................ucgguggUcuuucguucgcuu....................      12     1     Fm1
.......................................................................ucggugggacuuucguucgcGu....................      2      1     Fm1
.......................................................................ucggugggacuAucguucgcuu....................      2      1     Fm1
.......................................................................uUggugggacuuucguucgcuu....................      3      1     Fm1
```

Star Mature

```
acgaagcgcaagagaacaaaccaccuguugcgcccggucgaaagcuuccgccaaucgccgauaaaaauugaucggugggacuuucguucgcuugugaugggugguagaucaac
.......................................................................ucggugggacGuucguucgcuu....................    17      1    Fm1
.......................................................................ucggugggacuuucgCucgcuu....................    1       1    Fm1
.......................................................................ucggugggacuuucguucgcuu....................    13100   0    Fm1
.......................................................................ucggugggacuuucguAcgcuu....................    29      1    Fm1
.......................................................................ucggAgggacuuucguucgcuu....................    1       1    Fm1
.......................................................................Ncggugggacuuucguucgcuu....................    5       1    Fm1
.......................................................................ucggugggacuuucAuucgcuu....................    3       1    Fm1
.......................................................................ucgguggAacuuucguucgcuu....................    1       1    Fm1
.......................................................................ucggugggacuuucguucAcuu....................    1       1    Fm1
.......................................................................ucggugggacuuucguucgcuug...................    11      0    Fm1
.......................................................................ucggugggacuuucguucgcuuA...................    176     1    Fm1
.......................................................................ucggugggacuuucguucgcuuU...................    4437    1    Fm1
.......................................................................ucggugggacuuucguucgcuuC...................    11      1    Fm1
.......................................................................ucggugggacuuucguucgcuuAu..................    38      1    Fm1
.......................................................................ucggugggacuuucguucgcuuUu..................    815     1    Fm1
.......................................................................ucggugggacuuucguucgcuuCu..................    10      1    Fm1
........................................................................cggugggacuuucguucgcuu....................    3       0    Fm1
........................................................................cggugggacuGucguucgcuu....................    1       1    Fm1

.................................cAggucgaaagcuuccgccaauc.........................................................    1       1    Fm2
.................................ccggucgaaagcuuccgccaauc.........................................................    29      0    Fm2
......................................................................aucggugggaGuuucguucgcu.....................    1       1    Fm2
.......................................................................ucggugggacuuucguuc........................    2       0    Fm2
.......................................................................ucggugggacuuucguucA.......................    2       1    Fm2
.......................................................................ucggugggacuuucguucgc......................    172     0    Fm2
.......................................................................ucggugggacuuucguuGgc......................    2       1    Fm2
.......................................................................ucggugggacuuucguGcgc......................    3       1    Fm2
.......................................................................ucggugggacuuucguCcgc......................    2       1    Fm2
.......................................................................ucggugggacuuucguuAgc......................    3       1    Fm2
.......................................................................ucggugggacuuucguucgcA.....................    1       1    Fm2
.......................................................................uAggugggacuuucguucgcu.....................    1       1    Fm2
.......................................................................ucggugggacuCucguucgcu.....................    1       1    Fm2
.......................................................................ucggugggacAuucguucgcu.....................    1       1    Fm2
.......................................................................ucggugggacuuucguGcgcu.....................    1       1    Fm2
.......................................................................Ncggugggacuuucguucgcu.....................    1       1    Fm2
.......................................................................ucggugggUcuuucguucgcu.....................    2       1    Fm2
.......................................................................ucggugggacuuucguucgcu.....................    419     0    Fm2
.......................................................................ucggugggacuuucguuAgcu.....................    3       1    Fm2
.......................................................................ucUgugggacuuucguucgcu.....................    2       1    Fm2
.......................................................................ucggugggacuuucguAcgcu.....................    2       1    Fm2
.......................................................................ucggugggacuuucguuUgcu.....................    3       1    Fm2
.......................................................................ucggugggacuuucguucgcG.....................    17      1    Fm2
.......................................................................ucAgugggacuuucguucgcuu....................    1       1    Fm2
.......................................................................ucgguAggacuuucguucgcuu....................    2       1    Fm2
.......................................................................ucgguggCacuuucguucgcuu....................    1       1    Fm2
.......................................................................ucggugggCcuuucguucgcuu....................    1       1    Fm2
.......................................................................ucggugggacuuucUuucgcuu....................    1       1    Fm2
.......................................................................Ncggugggacuuucguucgcuu....................    2       1    Fm2
.......................................................................ucggugggacuuucguucgcGu....................    1       1    Fm2
.......................................................................ucggugggacuuucguuGgcuu....................    12      1    Fm2
.......................................................................uGggugggacuuucguucgcuu....................    1       1    Fm2
.......................................................................ucggugggacuuucguucgcuG....................    36      1    Fm2
.......................................................................uUggugggacuuucguucgcuu....................    1       1    Fm2
.......................................................................ucggugggacAuucguucgcuu....................    6       1    Fm2
.......................................................................ucggugggacuuucguucgAuu....................    2       1    Fm2
.......................................................................ucggugggacuuucgCucgcuu....................    1       1    Fm2
.......................................................................ucggugggUcuuucguucgcuu....................    1       1    Fm2
.......................................................................ucgguUggacuuucguucgcuu....................    1       1    Fm2
.......................................................................ucggugggacuuucguuAgcuu....................    23      1    Fm2
.......................................................................ucggugggacuuuAguucgcuu....................    2       1    Fm2
.......................................................................ucggugggacuuucguCcgcuu....................    8       1    Fm2
.......................................................................ucgguggAacuuucguucgcuu....................    1       1    Fm2
.......................................................................ucggugggacuuucguGcgcuu....................    8       1    Fm2
.......................................................................ucggugggacuuucguAcgcuu....................    7       1    Fm2
.......................................................................ucggCgggacuuucguucgcuu....................    1       1    Fm2
.......................................................................ucggugggacCuucguucgcuu....................    2       1    Fm2
.......................................................................ucggugggacGuucguucgcuu....................    2       1    Fm2
.......................................................................ucggugggacuuucguuUgcuu....................    17      1    Fm2
.......................................................................ucggugggacuuucguucgcuA....................    11      1    Fm2
.......................................................................ucggugUgacuuucguucgcuu....................    3       1    Fm2
```

Star Mature

acgaagcgcaagagaacaaaccaccuguugcgcccggucgaaagcuuccgccaaucgccgauaaaauugaucggugggacuuucguucgcuugugaugggugguagaucaac			
....ucggugNgacuuucguucgcuu....................	1	1	Fm2
....ucggugggacuuucguucgcuu....................	2592	0	Fm2
....ucggugCgacuuucguucgcuu....................	1	1	Fm2
....ucggugggacuuucguucgUuu....................	9	1	Fm2
....ucggugggacuuucguucgcuug...................	9	0	Fm2
....ucggugggacuuucguucgcuGg...................	1	1	Fm2
....ucggugggacuuucguuAgcuug...................	1	1	Fm2
....ucggugggacuuucguucgcuuU...................	1797	1	Fm2
....ucggugggacuuucguucgcuuA...................	38	1	Fm2
....ucggugggacuuucguucgcuuUu..................	563	1	Fm2
....ucggugggacuuucguucgcuuAu..................	51	1	Fm2
....cggugggacuuucguucgc......................	1	0	Fm2
....cggugggacuuucguucgcu.....................	1	0	Fm2
....ggugggacuuucguucgcu.....................	1	0	Fm2
....ggugggacuuucguucgcuuU...................	6	1	Fm2
